# Chloral Hydrate as a Vesicant

**Published:** 1885-10

**Authors:** A. M. Fauntleroy


### Chloral Hydrate as a Vesicant.

Powdered chloral sprinkled on adhesive plaster and melted by a gentle heat (not more than enough to cause the plaster to adhere to the flesh) is applied while warm to the part where the blister is wanted; within a few minutes a gentle heat is felt, increasing in intensity for a short time, then gradually easing off, and at the end of about ten minutes the part is free from pain. At the expiration of this time, or as soon as the pain has subsided, the plaster, if removed, will disclose a surface as effectually blistered as by a cantharidal plaster after six hours. Thus within about ten minutes the work of an old-fashioned blister is accomplished, with many advantages over the latter, (1) rapidity of action, (2) the ease of application, (3) the non-occurrence of strangury, and (4) farther, it may never be taken off to have the blister dressed, but may be allowed to remain until the plaster loosens and comes off itself, the blistered surface in the meanwhile healing kindly.—*A. M. Fauntleroy, M. D., in the Southern Clinic.*

